# Identification and Characterization of Circular Single-Stranded DNA Genomes in Sheep and Goat Milk

**DOI:** 10.3390/v13112176

**Published:** 2021-10-28

**Authors:** Marie-Thérèse König, Robert Fux, Ellen Link, Gerd Sutter, Erwin Märtlbauer, Andrea Didier

**Affiliations:** 1 Department of Veterinary Sciences, Faculty of Veterinary Medicine, Institute of Food Safety, Ludwig-Maximilians-Universität München, 85764 München, Germany; marie-therese.koenig@mh.vetmed.uni-muenchen.de (M.-T.K.); e.maertlbauer@mh.vetmed.uni-muenchen.de (E.M.); 2 Chair of Virology, Department of Veterinary Sciences, Faculty of Veterinary Medicine, Ludwig-Maximilians-Universität München, 80539 München, Germany; Robert.Fux@viro.vetmed.uni-muenchen.de (R.F.); Ellen.Link@viro.vetmed.uni-muenchen.de (E.L.); sutter@viro.vetmed.uni-muenchen.de (G.S.)

**Keywords:** BMMF, circular ssDNA, colon/breast cancer, *Cressdnaviricota*, *Genomoviridae*, milk, small ruminants

## Abstract

In recent years, a variety of circular replicase-encoding single-stranded (CRESS) DNA viruses and unclassified virus-like DNA elements have been discovered in a broad range of animal species and environmental samples. Key questions to be answered concern their presence in the human diet and their potential impact on disease emergence. Especially DNA elements termed bovine meat and milk factors (BMMF) are suspected to act as co-factors in the development of colon and breast cancer. To expand our knowledge on the occurrence of these potential pathogens in human nutrition, a total of 73 sheep and 40 goat milk samples were assayed by combining rolling circle amplification (RCA), PCR and Sanger sequencing. The present study further includes retail milk from the aforementioned species. We recovered 15 single stranded (ss) circular genomes. Of those, nine belong to the family *Genomoviridae* and six are members of the unclassified group of BMMF. Thus, dairy sheep and goats add to dispersal of CRESS viruses and circular ssDNA elements, which enter the food chain via milk. The presence of these entities is therefore more widespread in *Bovidae* than initially assumed and seems to be part of the common human nutrition.

## 1. Introduction

In recent years, a considerable number of small circular replicase-encoding single stranded DNA (CRESS) viruses [1,2,3] and unclassified virus-like DNA molecules [4,5,6] have been recovered from numerous animal species as well as from environmental samples. The *Cressdnaviricota* encompassing eight families represent a novel, rapidly growing phylum of these DNA viruses [7]. One of them—the *Genomoviridae*—comprises 10 genera isolated from all domains of live as well as from environmental samples [8]. The number of species within this family steadily increases and there are numerous uncultivated isolates in the databases, which are still unclassified. Their genomes are approximately 2–2.4 kb in length and encode at least two proteins, which are: (i) a rolling circle replication initiation protein (Rep) and (ii) a capsid protein (CP). Pairwise comparison of CP sequences revealed a higher degree of divergence compared to Rep. Assignment to a genus is therefore mainly based on the amino acid (aa) sequence of Rep [9]. These Rep proteins initiate rolling circle replication (RCR) of the viral genomes [10]. In addition to studies focussing on the molecular mechanism of replication, some studies describe the occurrence of circular single stranded (ss) DNAs in livestock and foods of animal origin. Investigations from China reported on the characterization of CRESS viral genomes from the genital tract and blood of cattle [11,12]. Viral genomes and CRESS DNAs have been found in various meat samples in Porto Alegre, Brazil and in San Francisco, USA. However, in these reports the detection was not linked to human diseases [13,14]. In contrast, multiple unclassified circular viral-like DNA elements, isolated from raw milk and cow milk at retail in Germany are suspected to contribute to the emergence of colon and breast cancer and neurodegenerative diseases [5,15,16,17]. These DNAs have been termed “BMMF” (bovine meat and milk factors) and are currently assigned to BMMF groups 1–4 according to their molecular characteristics [15]. Often a certain strain is synonymously described by abbreviations like HCBI (healthy cattle blood isolate) and CMI (cow milk isolate) thus pointing to the matrix from which it has been originally isolated. The majority of isolates is assorted to BMMF group 1 and 2, showing similarities to Sphinx 1.76 and Sphinx 2.36 DNA initially described by Manuelidis [4]. A few recovered isolates have been identified as genomoviruses and were assigned to BMMF group 3. Group 4 embodies a single isolate exhibiting similarities to a *Psychrobacter* sp. plasmid. BMMF group 1 and 2 members lack a CP and therefore differ significantly from group 3. All BMMF contain *rep*-genes and apparently replicate via RCR. In addition to a potential hairpin structure as a putative origin of replication, all BMMF1 genomes feature tandem repeats (TR) in proximity to *rep* [17]. These attributes underpin the theory on the phylogeny of circular ssDNAs, which are supposed to have evolved from bacterial plasmids via RNA virus gene transfer [18,19]. Therefore, borders between CRESS viruses, plasmids and phages sometimes blur.

From a disease related point of view, latest studies demonstrated the presence of BMMF encoded antigens in colorectal peritumor and tumor tissue and therefore support the idea of their involvement in colorectal cancerogenesis [20]. Thus far, the isolation of *Cressdnaviricota* and BMMF has been reported from meat, milk and dairy products of “taurine” cattle descending from the European aurochs. Despite the potential correlation between these DNAs and human cancer [21,22,23], data from other *Bovidae* are still missing. Beside “taurine” cattle, “zebuine” cattle, water buffaloes and small ruminants contribute to the worldwide milk supply [24]. In a recently published study, we showed the occurrence of circular ssDNA and a gemycircularvirus in water buffalo milk [25]. Data from studies focussing on domestic livestock are scarce and display a still narrow basis for a risk assessment concerning potential adverse effects on human health.

The present study highlights the recovery and characterization of circular DNA genomes in sheep and goat milk, thereby demonstrating a considerably more frequent occurrence of genomoviruses and BMMF in milk of dairy *Bovidae* than assumed so far.

## 2. Methods

### 2.1. Sample Collection

A total of 113 samples of sheep and goat milk were collected from eight different flocks in Germany. Five different sheep farmers contributed altogether 73 samples (flock A: *n* = 20, flock B: *n* = 15, flock C: *n* = 15, flock D: *n* = 3, flock E: *n* = 20) and three goat farmers added 40 further milk samples to the study (flock F: *n* = 4, flock G: *n* = 20, flock H: *n* = 16). Animals were kept in Hesse, Lower Saxony, North Rhine-Westphalia, Baden-Württemberg and Bavaria. After collection, that was carried out during the daily milking routine, the milk was cooled to 4 °C and cold-shipped to the Institute of Food Safety within 48 h. Additionally, commercially available sheep milk (*n* = 6) and goat milk (*n* = 6) produced in Germany, Austria and Spain was bought in German supermarkets.

### 2.2. DNA Extraction and RCA

DNA was extracted from 200 µL milk using the QIAamp DNA Mini Kit (Qiagen, Hilden, Germany) according to the manufacturer’s instructions. DNA concentration and purity (i.e. OD260/OD280 ratio) were determined on a DeNovix DS-11 FX (DeNovix, Wilmington, DE, USA) spectrophotometer. Rolling circle amplification (RCA) of approx. 40 ng DNA was performed by the TempliPhi Amplification Kit (Cytiva, Marlborough, MA, USA) with random primers according to the manufacturer’s instructions. Isothermal amplification time at 30 °C was set to 18 h.

### 2.3. Recovery of Viral-like DNA Elements

Initial screening of RCA products for the presence of Sphinx-like DNA included three primer pairs designed via the *rep*-gene of Sphinx 1.76 (GenBank Acc. No. HQ444404.1; accessed in September 2021) and one designed via the *rep*-gene of Sphinx 2.36 (GenBank Acc. No. HQ444405.1; accessed in September 2021). A similar approach included 13 primer pairs for detection of genomoviruses. All PCRs were run in a volume of 50 µL on a TProfessional Gradient 96 Thermocycler (Biometra, Jena, Germany). ThermoPrime Plus Polymerase (ThermoScientific, Waltham, MA, USA) at 3 U/reaction was applied. The final concentration of dNTPs (ThermoScientific, Waltham, MA, USA) of 200 µM each. Primer sequences as well as the individual amplification programs are summarized in Supplementary Table S1 (sheets 1 + 2). PCR products were purified from agarose gels with HighYield PCR Purification/Gel Extraction Kit (SLG, Südlaborbedarf, Gauting, Germany) and subjected to Sanger sequencing on both strands. MWG Eurofins (Ebersberg, Germany) operated all sequencing reactions performed in the present study. PCR screening sequencing results were used for inversed primer design to obtain full-length sequences of the circular DNA elements (Supplementary Table S1, sheet 3). Additional PCRs including abutting primers for amplification of BMMF2 sequences published by de Villiers et al. [15] and for BMMF1 sequences published by Whitley et al. [16] were carried out.

### 2.4. Cloning and Full-Length Sequencing

Inverse, gel-purified PCR products were ligated with pCR2.1-TopoTA vector (Invitrogen, Carlsbad, CA, USA) according to the manufacturer’s instructions. Afterwards, chemically competent *Escherichia coli* DH5 alpha were transformed and the resulting pellet was plated on LB-agar with ampicillin (100 µg/mL). Five clones from each plate were subjected to MiniPrep Plasmid isolation via GenElute Plasmid Miniprep Kit (Sigma Aldrich, Taufkirchen, Germany). After control digest of 500 ng plasmid DNA with EcoRI (New England BioLabs, Ipswich, MA, USA) insert-bearing clones were sequenced via the M13rev-29 and M13uni-21 primer binding sites present in the vector.

### 2.5. Data Analysis

All full-length sequences were subjected to BlastSearch against the nucleotide database at NCBI [26]. Multiple sequence alignments and phylogenetic trees were computed in MEGA software (v. 10.2.6) by the means of the Maximum likelihood method. Bootstrap values were computed with 500 replicates [27]. SDT v1.2 was utilised for displaying pairwise genome identity scores of novel genomoviruses calculated from pairwise alignments generated by MUSCLE [28]. Tandem repeats [29] and inverted repeats [30] upstream the *rep*-gene sequences were checked at Emboss explorer. Maximum size of tandem repeats was restricted to 30 nucleotides (nt) and the minimum size of inverted repeats was set to 4 nt. ORFfinder software (version 1.3.0) at NCBI [31] served for in silico detection of potential open reading frames. Illustrations were plotted with SeqBuilder Pro of the DNASTAR software (Lasergene Inc., v. 17.1. DNASTAR. Madison, WI, USA) [32]. ATG or TTG as alternative start codons were allowed. The minimum ORF size was limited to 75 amino acids (aa). Only ORFs ≥ 95 aa were included in further comparative and functional analyisis. Putative intron acceptor-/donor sites and conserved amino acid motifs were identified manually. All annotated genomes from this study were deposited in the GenBank database (Accession numbers OK148616-OK148630).

## 3. Results and Discussion

### 3.1. General Data Analysis and Interpretation

The present study aimed to detect and characterize circular single stranded DNAs (CRESS viruses and BMMF) in sheep and goat milk, thus expanding the current knowledge on the occurrence of these factors in animal-based foods. To cover this topic, individual milk samples from flocks kept in different German provinces as well as milk available at retail were assayed. Grocery milk packages originate from Germany, Austria and Spain. Table 1 summarizes the overall information on the origin and basic features of circular DNA elements detected.

Circular ssDNA elements that were detected in individual milk samples originated from two out of five sheep flocks. In flock B, 40% (6/15) of the sheep milk samples contained circular full-length sequences with one animal bearing two different sequences. In contrast, flock C comprised 13% (2/15) positive individual samples only. Results obtained from sheep and goat milk on the flock level thus markedly differ from the recently published data on water buffalo milk: (i) both water buffalo herds under study were affected and (ii) 56% of the animals from herd 1 and 42% from herd 2 tested positive for full-length sequences [25]. Although three out of 40 individual goat milk samples yielded amplicons of the expected size and sequence with at least one primer pair during PCR screening, we failed to retrieve full-length circular genomes from any of them. This discrepancy has already been observed when water buffalo milk was assayed [25] and again underlines the importance not to rely on the presence of sub-genomic amplicons to categorize an individual as positive. In contrast to the individual samples from the goat farms, four full-length genomes were recovered from goat milk at retail (GmGV9; GmI2, GmI5 and GmI6).

Overall, 50% of sheep and goat milk at retail contained either full-length CRESS-DNA or BMMF-like DNA. Interestingly, goat milk at retail tested positive although none of the individual samples under study contained circular ssDNA elements. Unfortunately, milk at the grocery cannot be traced back to the delivering farms especially those brands produced in Austria and Spain. To interpret this outcome, one has to keep in mind that due to blending at the dairy plant a milk package available at retail contains bulk milk from numerous individuals kept on different farms. Although the number of herds and animals assayed herein is far too low for a comprehensive prevalence estimation, a precautious interpretation of results could read as follows: There are positive and negative herds of sheep and goats kept in Germany. The percentage of affected animals in a positive herd is low to medium. Processing at the dairy plant leads to a medium percentage of packages positive for ssDNA elements at retail.

Furthermore, one has to consider that individual milk samples from the farms have been assayed as raw milk, whereas milk at retail has been pasteurized. As both sample types enabled the recovery of full-length ssDNA heat treatment at the dairy plant does not seem to negatively affect the isolation and detection procedure. Plenty other studies demonstrated the occurrence of CRESS-DNA in various samples like e.g., blood, feces, insects and plant material. Thus, a low-level contamination of milk with those entities cannot be fully excluded. Furthermore, one has to keep in mind, that all full-length genomes were recovered after RCA of input DNA. Thus, we assume that the “viral load” is rather low. A classical qPCR approach to determine viral copy number is questionable, because qPCR is based on the amplification of sub-genomic amplicons. In the present study, we occasionally faced diverging results after PCR screening and full-length amplification due to unknown reasons. Thus, a quantification of the sub-genomic fragments might be misleading. Future studies would benefit from a coherent definition of “BMMF/genomovirus positive” individuals and samples.

With regard to the cancer hypothesis one has to keep in mind that sheep and goats have a considerable impact on human nutrition worldwide [33,34]. Remarkably, small ruminants play a significant role in some Asian and African countries [35], like for example India, West and Central Africa, where we observe low colorectal cancer incidences [36].

### 3.2. Characterization of Genomoviral Sequences

**Figure 1:**
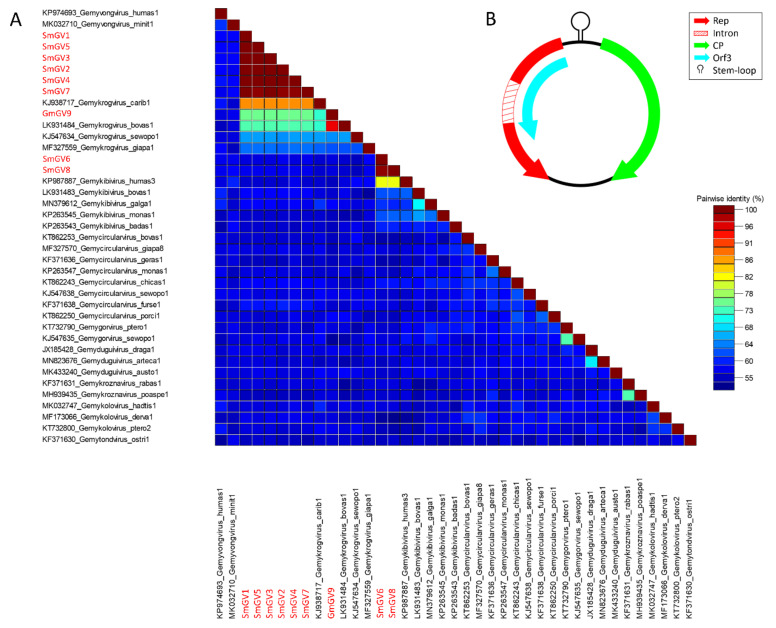
(**A**) Genome-wide pairwise nucleotide similarity score matrix including full-length genomoviruses from this study and representative members from nine genera of the family *Genomoviridae*. Sequences from this study are highlighted in red. (**B**) Schematic genome organization of the herein identified genomoviral sequences shows at least two major open reading frames and a stem-loop structure in the intergenic region.

**Figure 2:**
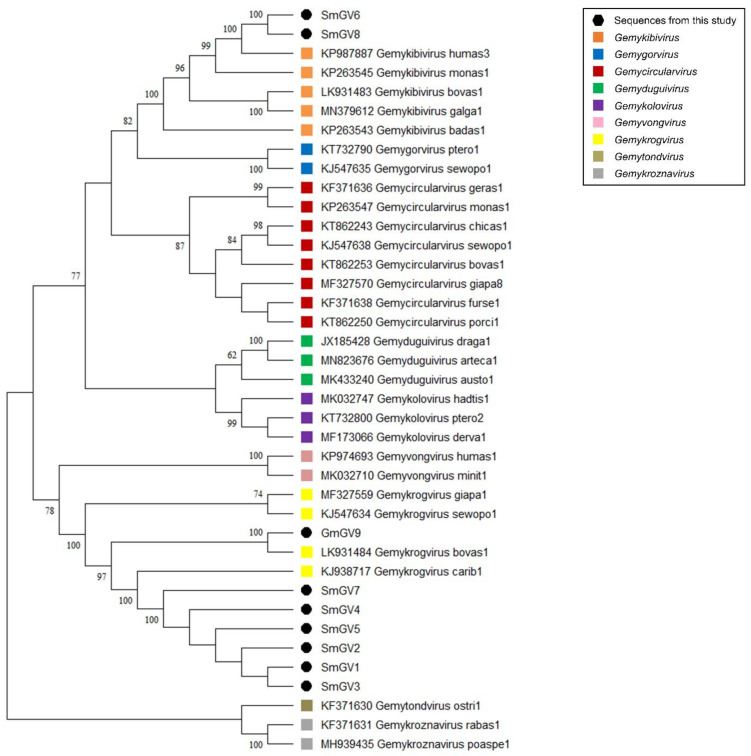
Maximum Likelihood phylogenetic tree of representative sequences from nine genomoviral genera and genomoviral sequences from this study. The evolutionary history was inferred by using the Maximum Likelihood method and Tamura-Nei model [37]. The bootstrap consensus tree inferred from 500 replicates is taken to represent the evolutionary history of the taxa analyzed [38]. Branches corresponding to partitions reproduced in less than 50% bootstrap replicates are collapsed. The percentage of replicate trees in which the associated taxa clustered together in the bootstrap test (500 replicates) are shown next to the branches [38]. Branch support values lower than 60% were not included. This analysis involved 39 nucleotide sequences. There were a total of 3641 positions in the final dataset. Evolutionary analyses were conducted in MEGA software. All taxa are indicated by name and the corresponding GenBank accession number.

In the present study, nine genomoviral sequences, eight from individual sheep milk samples and one from goat milk at retail, were detected. The isolates were termed sheep milk genomovirus 1–8 (SmGV1–8) and goat milk genomovirus 9 (GmGV9). Their length ranged from 2122 to 2192 nt. BlastSearch hits assigned the sequences to the family *Genomoviridae* (Supplementary Table S2, sheet 1). A genome-wide pairwise similarity comparison of the isolates from this study with representatives from nine genomoviral genera listed by the International Committee for Taxonomy of Viruses (ICTV) was performed to classify SmGV1–GmGV9 (Figure 1A). By setting the species demarcation threshold to 78% according to the suggestions given by Varsani and Krupovic in 2017 [9] the isolates SmGV1–5 and SmGV7 represent variants of the ICTV assigned species “*Gemykrogvirus carib1*” (KJ938717) isolated from caribou feces with 88% genome pairwise identity. These six isolates show >99% similarity among each other thus representing one strain. Isolate GmGV9 also fits the genus *Gemykrogvirus.* The highest sequence similarity (95%) is given to “*Gemykrogvirus bovas1*” (LK931484) which has been detected in taurine cattle serum. With 82% pairwise similarity, isolates SmGV6 and SmGV8 are variants of the species “*Gemykibivirus humas3*” (KP987887) and therefore belong to the genus *Gemykibivirus*. In order to obtain a depiction of results better comparable to the later presented BMMF isolates, a phylogenetic tree was calculated based on representative sequences from nine genomoviral genera and genomoviral sequences from this study (Figure 2).

In summary, seven out of nine genomoviral isolates under study cluster within species of the genus *Gemykrogvirus*, while the remaining two genomes belong to a species of genus *Gemykibivirus.* According to the similarity calculations and the prerequisites given by Varsani and Krupovic in 2017 [9], none of our isolates represents a novel species.

**Figure 3:**
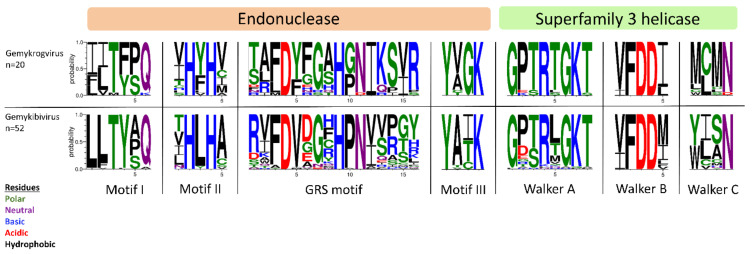
Illustration of amino acid variations in conserved Rep motifs using WebLogo3 [50]. Reps of ICTV-listed gemykrog- and gemykibiviruses as well as from the nine genomoviral isolates identified herein have been included in the alignment.

All isolates identified in this study exhibit typical features of *Genomoviridae* family members upon in silico analysis (Figure 1B). Beside a potential stem-loop structure at the origin of replication with a conserved nonanucleotide motif (TAATATTAT) at the loop-tip [39,40,41,42] genomes encode a Rep protein in complementary sense direction separated by a putative intron. These intron sequences have firstly been described in plant-infecting geminiviruses [43]. Like diverse ssDNA viruses, bacterial plasmids and phages, CRESS DNA viruses replicate through rolling circle replication (RCR) [44,45]. This mechanism is initiated by the Rep protein containing a distinct RCR endonuclease domain and a superfamily 3 (SF3) helicase domain [46]. Genomoviral Reps contain characteristic motifs associated with the endonuclease and helicase function. These motifs slightly differ between genera [9]. All conserved motifs identified in the new isolates are summarized in Table 2 and the variation in the amino acid motifs of all gemykibivirus- and gemykrogvirus-isolates including the ICTV-listed ones is illustrated in Figure 3. In addition to the genome-wide sequence comparison, amino acid motifs confirm the assignment of SmGV1–5, SmGV7 and SmGV9 to the genus *Gemykrogvirus* and SmGV6 and 8 to the genus *Gemykibivirus.* RCR motif I is thought to represent an essential part for recognition of the origin of replication and is located at the N-terminus of the Rep. RCR Motif II comprises two histidines ‘u-His-Tyr-His-u’ potentially coordinating metal ions that function as cofactors for endonuclease activity (u denotes hydrophobic residues). The third RCA motif seems to play an important role in dsDNA cleavage, covalent attachment of Rep and positioning during catalysis. GRS motifs separating motifs II and III have been identified for the first time in members of the family *Geminiviridae* by Nash et al. [47]. Their presence in isolates from this study indicates an involvement in the replication mechanism of genomoviruses. Furthermore all nine isolates exhibit typical SF3 helicase motifs (Walker A, B and C), which are necessary for dsDNA intermediate unwinding [48,49]. Interestingly, isolate SmGV2 contains a modified Walker C motif were a neutral amino acid (N) is replaced by an acidic one (D). Whether this substitution has a functional influence on the helicase activity has to be assayed. However, according to Varsani and Krupovic, 2017 [9] an acidic amino acid at the end of Walker C does not occur in any other genomoviral sequence.

In addition to the species assignment based on a genome-wide comparison, a further approach is based on the analysis of the Rep amino acid sequences. Results might slightly differ from whole genome analysis because of intra-familiar recombination [51]. In terms of the sequences retrieved herein, Rep comparison did not differ from the genome-wide assessment and thus did not hint to recombination events (Supplementary Figure S1A). Genomoviruses further encode CPs, which in general exhibit higher sequence divergence than Reps. This divergence can be seen in a similarity score matrix based on amino acid sequences of CPs (Supplementary Figure S1B). While the percentage identities of CPs from SmGV1–5, SmGV7 and SmGV9 to those from other gemykrogviruses are still higher than 83%, CPs from SmGV6 and SmGV8 share 72% similarity to their closest relative “Gemykibivirus humas3” only. BlastSearch hits for Rep, CP and putative ORF3 translation products are summarized in Supplementary Table S2 (sheets 2–4).

### 3.3. Characterization of BMMF-Related Sequences

**Figure 4:**
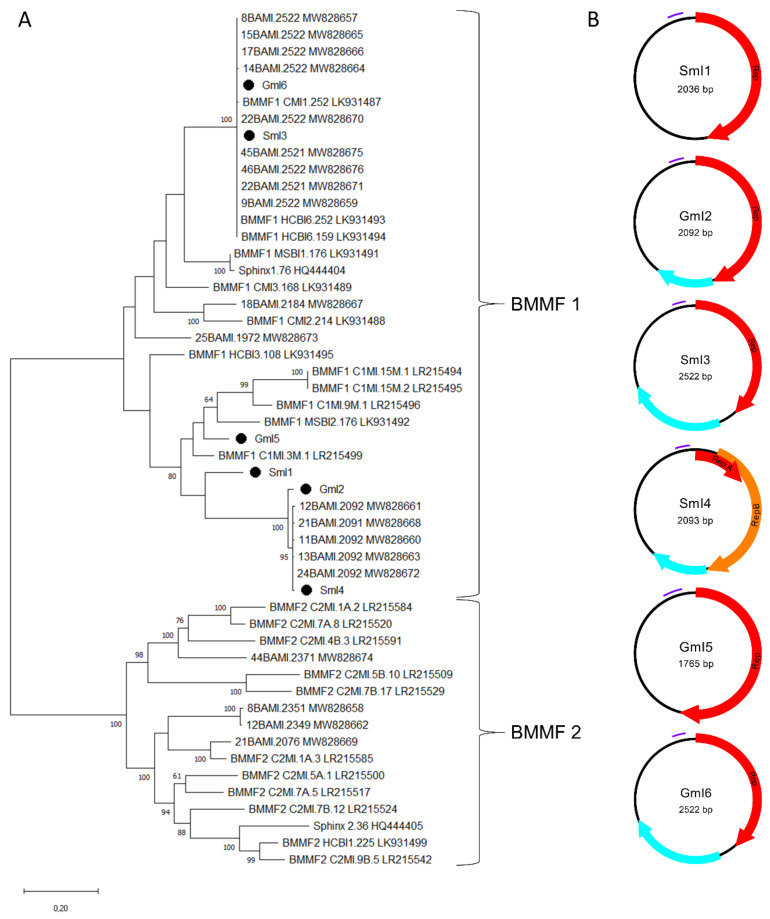
(**A**) Maximum Likelihood phylogenetic tree of novel full-length Sphinx-like DNA genomes, Sphinx 1.76, Sphinx 2.36, selected members of BMMF group 1 and 2 and recently published Sphinx-like DNA genomes isolated from water buffalo milk. The evolutionary history was inferred by using the Maximum Likelihood method and Tamura-Nei model [37]. The tree with the highest log likelihood (“-“50216.13) is shown. The percentage of trees in which the associated taxa clustered together is shown next to the branches [38]. Branch support values lower than 60% were not included. The tree is drawn to scale, with branch lengths measured in the number of substitutions per site. This analysis involved 51 nucleotide sequences. There were a total of 4500 positions in the final dataset. Black dots mark sequences found in this study. All taxa are indicated by name followed by the corresponding GenBank accession number. (**B**) Genomic organization of the six new BMMF-like genomes from this study. Red and orange arrows indicate *rep*-genes, blue arrows show further potential ORFs and purple bars represent tandem repeat regions.

In addition to nine genomoviral sequences, we retrieved another six circular ssDNA elements. Of those, three were recovered from sheep milk (two from milk at retail, one from herd C) and three from goat milk at retail. According to the BlastSearch hits, all reveal similarities to already published BMMF elements (Supplementary Table S2, sheet 1). A phylogenetic tree based on present sequences, previously published sequences found in water buffalo milk, BMMF from database entries, Sphinx 1.76 and Sphinx 2.36 is depicted in Figure 4A.

SmI3 and GmI6 belong to a cluster containing water buffalo milk isolates and other well characterized BMMF, such as CMI1.252 and HCBI6.252 (Figure 4A). GmI2 and SmI4 built a cluster with five sequences recently detected in water buffalo milk. SmI1 is only distantly related to the latter cluster. GmI5 is the most remote of our sequences showing relatedness only to BMMF1 isolates MSBI2.176, C1MI.9M.1 and C1MI.15M.1. Results depicted in the phylogenetic tree are consistent with the BlastSearch hits summarized in Supplementary Table S2 (sheet 1). Open reading frame prediction revealed the common presence of a *rep*-gene (Figure 4B). SmI4 bears a first short and a second larger *rep* that starts on ‘ATG’ or ‘TTG’ respectively. BlastSearch of in silico translated Reps showed highest similarities to BMMF1 or *Acinetobacter* sp. replication proteins (Supplementary Table S2, sheet 3). SmI1 and GmI5 are the only sequences lacking an additional, putative ORF.

All sequences from the present study could be assigned to the BMMF1 group according to their features. This group is mainly distinguishable from BMMF2 by the presence of a tandem repeat region that is present in the sheep and goat milk isolates as shown in Table 3. Tandem repeats that are typically located upstream the *rep*-gene possibly function as replicase recognition sites. The RCR mechanism of these DNA elements is further supported by inverted repeats, which are located 49–56 nt upstream the tandem repeat region. All BMMF1-related isolates exhibit such a palindromic sequence similar to a motif in BMMF1 elements published by zur Hausen et al. [17].

## 4. Conclusions

Results recorded in the present study substantiate our hypothesis that the occurrence of Cressdnaviricota and BMMF-related DNA elements in milk is not as narrowly restricted to “taurine” cattle as previously assumed. Sheep and goats kept for dairy purposes also shed those entities with milk. Thus, the present study contributes to the growing body of evidence on ssDNA elements frequently detectable in food of animal origin. From an epidemiological point of view, latest data published by Lechmann and colleagues in 2021 support the idea of interindividual and even interspecies transmission for gemykrogviruses [52]. Due to the marked discrepancies between the occurrence of viral DNA elements on herd level and in grocery packages an in depth prevalence assessment is needed. These future investigations should also include putative environmental CRESS sources like forage or water supply that might cause an affection of dairy ruminants and thus milk. Knowledge on affection levels of flocks and individuals could provide the first step for implementation of potential countermeasures. With regard to the circular ssDNA linked cancer hypothesis, further research efforts on underlying biological mechanisms and risk assessment are urgently needed.

## Figures and Tables

**Table 1:** Overview of all circular genomes detected in this study with accession numbers, sequence length, DNA-types, species and origin of isolation. Interestingly, two genomoviral sequences were isolated from sheep No. 115.

Isolate	Accession Number	Length in Nucleotides (nt)	DNA-Type	Species	Origin
SmGV1	OK148616	2191	Genomovirus	Sheep No. 109	Flock B
SmGV2	OK148617	2192	Genomovirus	Sheep No. 110	Flock B
SmGV3	OK148618	2190	Genomovirus	Sheep No. 111	Flock B
SmGV4	OK148619	2190	Genomovirus	Sheep No. 113	Flock B
SmGV5	OK148620	2191	Genomovirus	Sheep No. 115	Flock B
SmGV6	OK148621	2124	Genomovirus	Sheep No. 115	Flock B
SmGV7	OK148622	2190	Genomovirus	Sheep No. 117	Flock B
SmGV8	OK148623	2125	Genomovirus	Sheep No. 151	Flock C
GmGV9	OK148624	2122	Genomovirus	Goat	Retail
SmI1	OK148625	2036	Sphinx 1.76-like (BMMF1)	Sheep No. 153	Flock C
GmI2	OK148626	2092	Sphinx 1.76-like (BMMF1)	Goat	Retail
SmI3	OK148627	2522	Sphinx 1.76-like (BMMF1)	Sheep	Retail
SmI4	OK148628	2093	Sphinx 1.76-like (BMMF1)	Sheep	Retail
GmI5	OK148629	1765	Sphinx 1.76-like (BMMF1)	Goat	Retail
GmI6	OK148630	2522	Sphinx 1.76-like (BMMF1)	Goat	Retail

Abbreviations: SmGV—sheep milk genomovirus; GmGV-goat milk genomovirus; SmI-sheep milk isolate: GmI–goat milk isolate.

**Table 2:** Summary of conserved motifs in genomoviral sequences after intron clearing and in silico translation.

Isolate	Nonanucleotide	Motif I	Motif II	GRS Motif	Motif III	Walker A	Walker B	Walker C
SmGV1	TAATATTAT	IITFPQ	VHYHV	TAFDYFGAHGNIKSVR	YVGK	GPTRTGKT	VFDDI	MCMN
SmGV2	TAATATTAT	IITFPQ	VHYHV	TAFDYFGAHGNIKSVR	YVGK	GPTRTGKT	VFDDI	MCMD
SmGV3	TAATATTAT	IITFPQ	VHYHV	TAFDYFGAHGNIKSVR	YVGK	GPTRTGKT	VFDDI	MCMN
SmGV4	TAATATTAT	IITFPQ	VHYHV	TAFDYFGAHGNIKSVR	YVGK	GPTRTGKT	VFDDI	MCMN
SmGV5	TAATATTAT	IITFPQ	VHYHV	TAFDYFGAHGNIKSVR	YVGK	GPTRTGKT	VFDDI	MCMN
SmGV6	TAATATTAC	LFTYSQ	THLHA	RKFDVVGFHPNIISTI	YATK	GPSRTGKT	VFDDI	WLSN
SmGV7	TAATATTAT	IITFPQ	VHYHV	TAFDYFGAHGNIKSVR	YVGK	GPTRTGKT	VFDDI	MCMN
SmGV8	TAATATTAC	LFTYSQ	THLHA	RKFDVEGFHPNIISTI	YATK	GPSRTGKT	VFDDI	WLSN
GmGV9	TAATATTAT	IIMFPQ	IHYHI	TAFDYFGAHGNIKSIR	YVGK	GPTRTGKT	VFDDI	MCMN

**Table 3:** BMMF-like DNA isolates and inherent tandem repeat regions.

Isolate	Period Size	Copy Number	Sequence	Nt between TR and RepA	Nt between TR and RepB
SmI1	22	3.5	CCTACGTTTACCCATCAATACC	60	-
GmI2	22	3.5	ACACCGTTTACCCATCAATATG	59	-
SmI3	22	3.8	ATACCCCTACGTTTACCGATCA	60	-
SmI4	22	3.5	CACCGTTTACCCATCAATATGA	27	151
GmI5	22	3.8	ATACTCCTAGGTTTACCTACCA	59	-
GmI6	22	3.8	ATACCCCTACGTTTACCGATCA	60	-

## Data Availability

All annotated genomes from this study were deposited in the GenBank database (Accession numbers OK148616-OK148630).

## Supplementary Materials

The following are available online at https://www.mdpi.com/article/10.3390/v13112176/s1, Figure S1: SDT-matrices of Reps and CPs, Table S1: Primer-sets, Table S2: BlastSearch hits.
