# Supplementary material for: Identification and Characterization of Circular Single-Stranded DNA Genomes in Sheep and Goat Milk
Source: Viruses. 2021 Oct 28;13(11):2176. doi: 10.3390/v13112176 (PMC8621823; doi:10.3390/v13112176)
Supplement: Supplementary file 1 [file viruses-13-02176-s001.zip › Supplementary Figure S1.pdf]

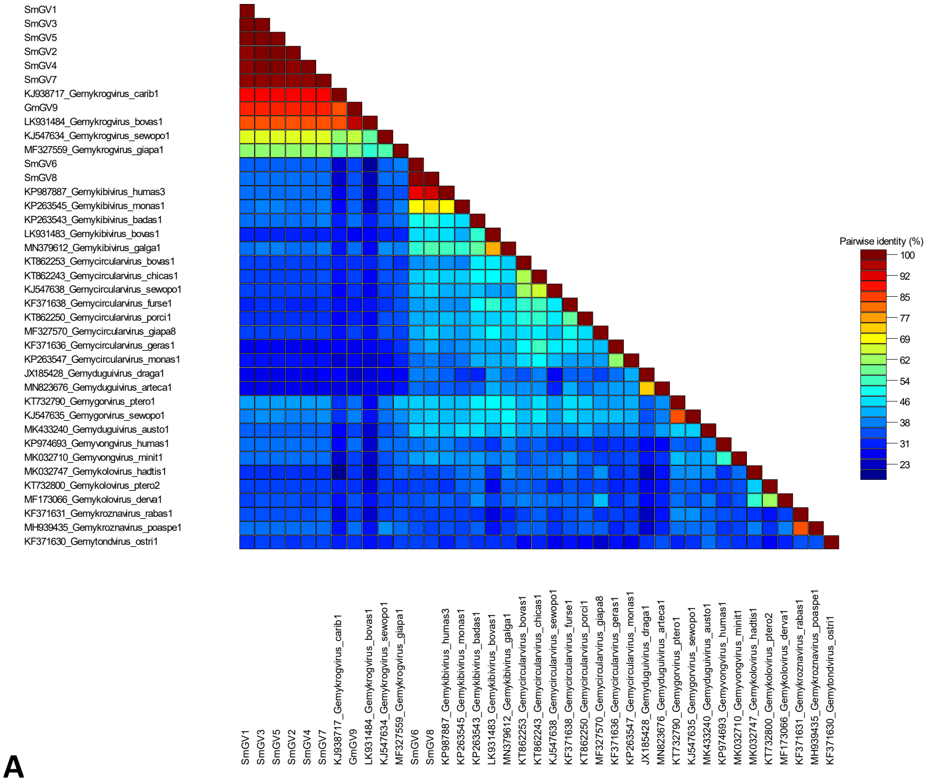

A

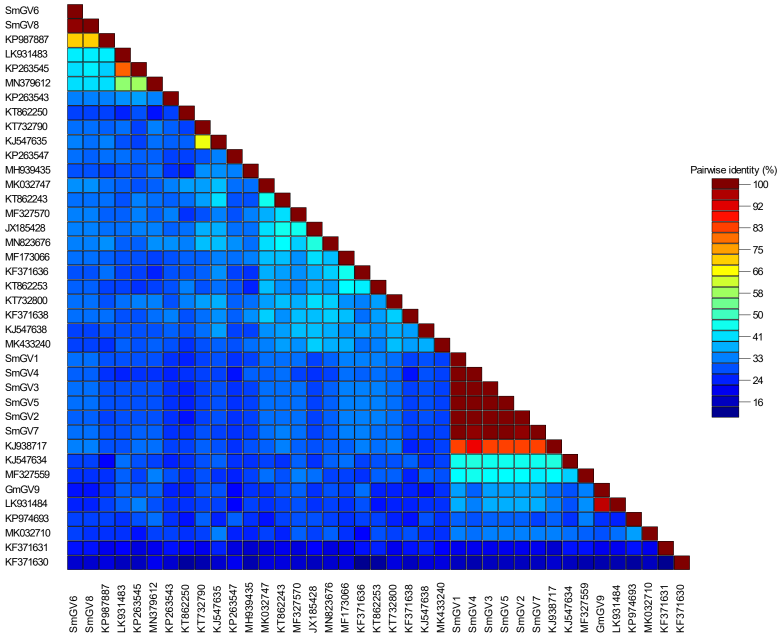

B

Supplementary Figure S1. Genome-wide pairwise amino acid similarity score matrices including Reps **(A)** and CPs **(B)** of all genomoviruses from this study and representative members from nine genera of the family *Genomoviridae* using SDT v1.2.
